# The Role of Non-Coding RNAs in the Sorafenib Resistance of Hepatocellular Carcinoma

**DOI:** 10.3389/fonc.2021.696705

**Published:** 2021-07-22

**Authors:** Xinyao Hu, Hua Zhu, Yang Shen, Xiaoyu Zhang, Xiaoqin He, Ximing Xu

**Affiliations:** ^1^Department of Oncology, Renmin Hospital of Wuhan University, Wuhan, China; ^2^Cancer Center, Renmin Hospital of Wuhan University, Wuhan, China; ^3^Department of Neurosurgery, Renmin Hospital of Wuhan University, Wuhan, China

**Keywords:** hepatocellular carcinoma, sorafenib resistance, microRNA, long non-coding RNA, circular RNA

## Abstract

Hepatocellular carcinoma (HCC) is the second most common cause of cancer-related death. Sorafenib is approved by the U.S. Food and Drug Administration to be a first-line chemotherapy agent for patients with advanced HCC. A portion of advanced HCC patients can benefit from the treatment with sorafenib, but many patients ultimately develop sorafenib resistance, leading to a poor prognosis. The molecular mechanisms of sorafenib resistance are sophisticated and indefinite. Notably, non-coding RNAs (ncRNAs), which include long ncRNAs (lncRNAs), microRNAs (miRNAs) and circular RNAs (circRNAs), are critically participated in the occurrence and progression of tumors. Moreover, growing evidence has suggested that ncRNAs are crucial regulators in the development of resistance to sorafenib. Herein, we integrally and systematically summarized the molecular mechanisms and vital role of ncRNAs impact sorafenib resistance of HCC, and ultimately explored the potential clinical administrations of ncRNAs as new prognostic biomarkers and therapeutic targets for HCC.

## Background

Hepatocellular carcinoma (HCC) is the paramount form of primary liver cancer, accounting for 75-85% (1). And it is deemed as the second leading cause of cancer-related deaths worldwide, with high probability of metastasis and lethality. Hepatitis B virus (HBV) or hepatitis C virus (HCV) infection, which stimulates chronic inflammation in the liver, is considered to be a dominating risk factor of HCC (2). Meaningfully, detecting early and taking treatment timely can obtain relatively long survival in HCC patients. However, patients are often diagnosed at an advanced stage because of the complex etiology, latent onset, difficulty in diagnosis and rapid progression of HCC. At this stage, chemotherapy is a major available palliative treatment (3), but chemotherapeutic drugs are prone to develop resistance during the course of HCC treatment.

Sorafenib is a first-line chemotherapeutic agent approval by the US Food and Drug Administration to treat the advanced HCC through inhibiting angiogenesis and cell proliferation (4). As a kinase inhibitor of multiple targets, it is capable of blocking tumor cell multiplication *via* restraining the activation of Raf-1, B-RAF and kinases in Ras/Raf/MEK/ERK pathway. In addition, sorafenib inhibits the generation of tumor blood vessels *via* regulating platelet-derived growth factor receptor (PDGFR-β), Fms-like tyrosine kinase (FLT-3), hepatocyte factor receptor (C-Kit), vascular endothelial growth factor receptor (VEGFR) 2, VEGFR-3 and other tyrosine kinases (5). However, patients are vulnerable to develop resistance to sorafenib during treatment, resulting in poor outcomes, so further studies are needed to investigate the precise mechanisms of sorafenib resistance (6).

**Figure 1:**
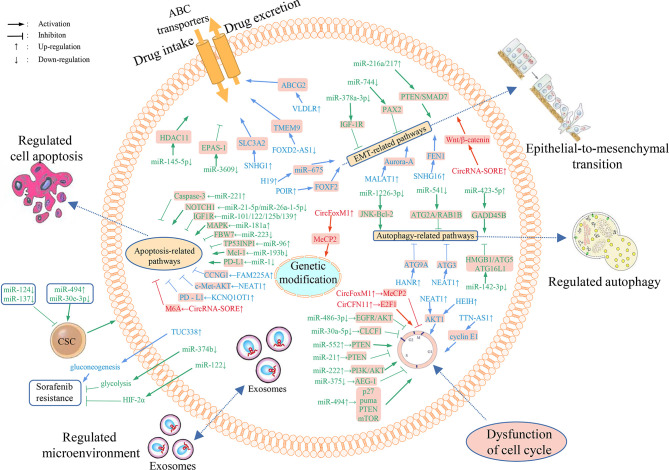
Overview of the mechanisms of ncRNAs involved in HCC resistance to sorafenib and dysregulated ncRNAs’ differential expression in sorafenib resistant HCC cells. Numerous miRNAs, lncRNAs and circRNAs are implicated in sorafenib resistance through regulating the expression of downstream target genes and affecting drug transport, metabolism, cell multiplication, autophagy, apoptosis, cell cycle, EMT, tumor microenvironment, and gene modifications.

Only 2% of human genome is composed of protein-coding sequences, the rest are non-coding sequences (7). Non-coding RNAs (ncRNAs) principally consist of miRNAs, lncRNAs and circRNAs, which are widely associated with the transcription and post-transcription regulation, and also exert a critical action in the occurrence and development of cancer, as well as resistance of sorafenib (8). Herein, we discussed the molecular mechanisms of sorafenib resistance and the role of ncRNAs in this process (**Figure 1**), thus providing new ideas to antagonize sorafenib resistance, enhance the efficacy of sorafenib, and improve the outcome of HCC patients.

## Mechanisms of Sorafenib Resistance in HCC

Some individuals with HCC do not respond to sorafenib in clinical practice, and even if some patients do respond initially, they quickly become refractory (9), mainly due to the primary and acquired resistance to sorafenib. The primary mechanisms of the resistance to sorafenib are summarized here, more details are given in **Tables 1**, **2**.

**Table 1:** miRNAs and sorafenib resistance in HCC.

miRNAs	Expression	Effects on sorafenib resistance	Target	Mechanism	References
miR-423-5p	up-regulated	Inhibiting	GADD45B	autophagy	(10, 11)
miR-142-3p	down-regulated	Promoting	HMGB1, ATG5, ATG16L1	autophagy	(12, 13)
miR-221	up-regulated	Promoting	Caspase-3	apoptosis	(14)
miR-541	down-regulated	Inhibiting	ATG2A/RAB1B	autophagy	(15)
miR-30e-3p	down-regulated	Promoting	TP53/MDM2, EpCAM, PTEN, p27	CSCs	(16)
miR-486-3p	down-regulated	Inhibiting	FGFR4, EGFR	AKT activation	(17)
miR-122	down-regulated	Inhibiting	SERPINB3	HIF-2α/CSCs	(18, 19)
miR-30a-5p	down-regulated	Inhibiting	CLCF1	AKT activation	(20)
miR-1226-3p	down-regulated	Inhibiting	DUSP4	JNK-Bcl-2 axis	(21)
miR-552	up-regulated	Promoting	PTEN	AKT activation	(22)
miR-124	down-regulated	Inhibiting	CAV1	CSCs	(23)
miR-181a	up-regulated	Promoting	RASSF1	MAPK	(24)
miR - 222	up-regulated	Promoting	PI3K/AKT	AKT activation	(25)
miR-378a-3p	down-regulated	Inhibiting	IGF-1R	EMT	(26)
miR-744	down-regulated	Inhibiting	PAX2	EMT	(27)
miR-374b	down-regulated	Inhibiting	hnRNPA1/PKM2	glycolysis	(28)
miR-21	up-regulated	Inhibiting	PTEN	AKT activation	(29, 30)
miR-375	down-regulated	Inhibiting	AEG-1	AKT activation	(31)
miR-223	down-regulated	Promoting	FBW7	apoptosis	(32)
miR-145-5p	down-regulated	Inhibiting	HDAC11	metabolism	(33)
miR-96	up-regulated	Promoting	TP53INP1	apoptosis	(34)
miR-494	up-regulated	Promoting	p27, puma, PTEN, mTOR	AKT, CSCs	(35, 36)
miR-21-5p	down-regulated	Inhibiting	EZH2	NOTCH1	(37)
miR-26a-1-5p	down-regulated	Inhibiting	EZH2	NOTCH1	(37)
miR-3609	down-regulated	Inhibiting	EPAS-1	metabolism	(38)
miR-101/122/125b/139	up-regulated	Promoting	IGF1R	apoptosis	(39)
miR-193b	down-regulated	Inhibiting	Mcl-1	apoptosis	(40)
miR-1	down-regulated	Inhibiting	PD-L1	apoptosis	(41)
miR-137	down-regulated	Inhibiting	ANT2	CSCs	(42)
miR-216a/217	up-regulated	Promoting	PTEN, SMAD7	EMT	(43)

**Table 2:** lncRNAs and sorafenib resistance in HCC.

lncRNAs	Expression	Effects on sorafenib resistance	Interact with	Target	Mechanism	References
**SNHG1**	up-regulated	Promoting	miR-21	SLC3A2	transfer	(44)
**HANR**	up-regulated	Promoting	miR-29b	ATG9A	autophagy	(45)
**FAM225A**	up-regulated	Promoting	miR-130a-5p	CCNG1	apoptosis	(46)
**NEAT1**	up-regulated	Promoting	miR-149-5p	AKT1	Akt activation	(47)
**HEIH**	up-regulated	Promoting	miR-98-5p	PI3K/AKT	Akt activation	(48)
**SNHG16**	up-regulated	Promoting	miR-140-5p	FEN1	EMT	(49, 50)
**KCNQ1OT1**	up-regulated	Promoting	miR - 506	PD - L1	apoptosis	(51)
**MALAT1**	up-regulated	Promoting	miR-140-5p	Aurora-A	EMT	(52, 53)
**H19**	up-regulated	Promoting	miR-675	miR - 675	EMT	(54)
**FOXD2-AS1**	down-regulated	Inhibiting	miR-150-5p	TMEM9	transfer	(55)
**TTN-AS1**	up-regulated	Promoting	miR-16-5p	cyclin E1	Akt activation	(56)
**NEAT1**	up-regulated	Promoting	miR-204 miR-335 miR-149-5p	ATG3 c-Met-AKT	autophagy apoptosis AKT activasion	(57, 58)
**POIR**	up-regulated	Promoting	miR-182-5p	FOXF2	EMT	(59)
**TUC338**	up-regulated	Promoting	**/**	RASAL1	gluconeogenesis	(60, 61)
**VLDLR**	up-regulated	Promoting	**/**	ABCG2	transfer	(62)

### Primary Resistance

The primary resistance of HCC to sorafenib, existing prior to drug treatment, is attributed mainly to the genetic heterogeneity (63), while the precise mechanism is still unclear. Epidermal growth factor receptor (EGFR), is the most well-studied target related to the primary resistance to sorafenib. EGFR, located at the surface of epithelial cells, when bound to its ligands, can result in the activation of downstream pathways, thereby regulating cell proliferation (64). Over half of the patients diagnosed with HCC have aberrant EGFR/HER3 activation and over-expression of EGFR and its ligands (particularly the dual regulated ligands), which suppress the antitumor capacity of sorafenib. The efficacy of sorafenib can be improved when sorafenib combined with RNA interference agents inhibiting EGFR/HER-3 phosphorylation. Studies suggested that activated EGFR may be a promising predictor of primary sorafenib resistance in HCC cells, and overexpression of EGFR or its ligands may result in continuous activation of EGFR downstream pathways and subsequent resistance to sorafenib (65). Additionally, another downstream pathway of EGFR, the Ras/Raf/MEK/ERK pathway, is activated in sorafenib-resistant patients, further corroborating the vital action of EGFR in sorafenib resistance. The down-regulation of pERK may contribute to sorafenib resistance.

In addition, mitogen-activated protein kinase (MAPK) levels affect the susceptibility of HCC cells to sorafenib. Recent research has also demonstrated that c-Jun N-terminal kinase (JNK), another member of MAPK family, has great potential to predict the sorafenib sensitivity (65). Furthermore, sestrin2 (SESN2), an important component of the sestrin stress‐inducible protein family, participates in tumorigenesis and development by regulating a variety of downstream pathways, among which MAPK and AKT are closely associated with cell multiplication and metabolism (66). Upregulation of SESN2 confers primary resistance to sorafenib in HCC cells by activating AKT (67). Additionally, HCC patients with overexpressed VEGFA are significantly susceptible to sorafenib. VEGFA can stimulate stromal cells to secrete hepatocyte growth factor to induce tumor progression, suggesting that VEGFA may be a promising predictor of response to sorafenib in patients with HCC (68).

In summary, it is highly prospective to further explore the mechanism of primary resistance to sorafenib, identify biomarkers that predict sorafenib sensitivity, and then personalize the therapy for patients with different sensitivities to save the economic and time costs of ineffective treatment. It is also helpful in seeking new therapeutic targets and exploring novel strategies to combine sorafenib with other targeted drugs for more effective treatment.

### Acquired Resistance

Acquired sorafenib resistance can be induced under a variety of conditions, including reduced drug intake, enhanced intracellular drug metabolism, increased excretion, changes in molecular targets affecting the activation/inactivation of pathways, changes in DNA repair mechanisms, dysfunction of cell cycle-related proteins and tumor microenvironment regulation (69). Here we summarized several of the latest and most recognized mechanisms of acquired sorafenib resistance.

#### The Solute Carrier (SLC) Family

The human SLC superfamily transporter plays an important role in sorafenib uptake. Previous study has shown that SLC22 (organic cationic/anion transporter) is down-regulated in HCC and is closely related to sorafenib resistance. Moreover, down-regulated expression of SLC22A1 in human HCC is related to its DNA methylation (70). Therefore, demethylation agents targeting SLC22A1 methylation are promising for the treatment of patients resistant to sorafenib (71).

#### ATP Binding Box (ABC) Transporters Family

Removal of drugs from the cytoplasm is an important method of drug resistance. The ABC transporter is one of the largest family of membrane transporters. The genetic variants of ABC transporter genes, such as the ABCB family, the ABCC family and the ABCG2 family is related to clinical chemotherapy resistance (72). An *in vitro* research illustrated that the accumulation of sorafenib was lower in cells with ABCC2 overexpression than in cells with normal ABCC2 expression, suggesting that sorafenib resistance may be related to ABCC2 (MRP2) variation. Furthermore, downregulation of ABCC2 expression has the potential to restore sensitivity to sorafenib (73).

#### EGFR

EGFR/HER3 activation is associated with both primary and acquired drug resistance (74). The combination of gefitinib, which can down-regulate EGFR/HER3 expression, with sorafenib has been shown to increase tumor inhibition and prevent sorafenib resistance, demonstrating the role of EGFR/HER3 inhibition in the HCC treatment.

#### AKT Activation

The sustained action of sorafenib induces AKT activation, which further leads to resistance of HCC cells to sorafenib. AKT inhibition reverses sorafenib resistance by converting protective cellular autophagy into a cell-death mechanism. GDC0068, a new ATP competitive inhibitor of pan-AKT, acts synergistically with sorafenib in inhibiting the development of sorafenib-resistant HCC. Moreover, the combination of sorafenib with arsenic trioxide (ATO), an AKT-inhibited anti-cancer agent, enhances the anticancer activity of sorafenib against HCC (75). Furthermore, it has been found that the application of hepatocyte growth factor (HGF) in sorafenib treated HCC cells can activate the proto-oncogene MET, re-stimulate the downstream AKT and extracellular regulated protein kinases (ERK1/2) pathways, therefore inhibiting apoptosis. HCC cells treated by HGF can also induce the expression of early growth response protein (EGR1), which has strong correlation with sorafenib resistance. Taken together, HGF induced MET activation promotes the sorafenib resistance in HCC *via* AKT/ERK1/2-EGR1 pathway (76).

#### Hypoxia-Inducible Factors (HIF)

In patients continuously exposed to sorafenib, the anti-angiogenic effect of sorafenib causes a decrease in microvascular density, leading to intracellular hypoxia and favoring the selection of resistant cells adapted to the hypoxic microenvironment. Clinical studies suggest that overexpression of HIF-1 and HIF-2 in HCC patients is a reliable marker of poor prognosis (77). Therefore, sorafenib combined with HIF inhibitor therapy is a potential approach to overcome sorafenib resistance. It is important to note that HIF-1 and HIF-2 compensate for each other, and the removal of one HIF-subtype increases the expression of the other HIF-subtype. Hence, targeting both HIF-1 and HIF-2 is more effective.

#### Metallothionein (MT) -1G

Sorafenib activates the transcription factor nuclear factor erythrocyte 2-related factor 2, thus inducing the MT-1G expression. Downregulation of MT-1G was shown to improve the anticancer action of sorafenib both *in vitro* and *in vivo*. Knockdown of MT-1G by RNA interference increased the glutathione consumption and the lipid peroxidation, resulting in sorafenib-induced ferroptosis, which is a novel approach to regulate cell death (78). In summary, MT-1G may be a key regulator of sorafenib-resistant cells in human HCC and is a hopeful therapeutic target.

#### Cancer Stem Cells (CSC)

Tumorigenicity and chemoresistance of tumor cells can be increased by the acquisition of CSC characteristics. CD13, CD24, CD44, CD90, CD133 and EpCAM are potential markers for the enrichment of CSCs in HCC. CD133^+^ cells activate the AKT/PKB axis and Bcl-2 cell survival response, thus promoting chemoresistance (79). CD44 also contributes to the formation of sorafenib resistance and can serve as a predictor of sorafenib efficacy. Moreover, Nanog is a gene essential for self-renewal of CSCs in HCC. It has been shown that in sorafenib-resistant HCC cells, Nanog expression is promoted by the destabilization and significant downregulation of the transactivation response element RNA binding protein 2, as a result of autophagy-lysosomal protein hydrolysis, promoting the sorafenib resistance of HCC cells (80). In addition, the interaction between cyclin-dependent kinases 1 (CDK1) and the pluripotent transcription factor octamer binding transcription factor 4 has a crucial action in differentiation of embryonic stem cells. It has revealed that blocking the CDK1/PDK1/β-catenin pathway by the CDK1 inhibitor RO3306 improves the therapeutic efficacy of sorafenib in a preclinical HCC model (81). Overall, CSCs-based studies are expected to reverse resistance to sorafenib and improve its efficacy.

#### Activator of Thyroid and Retinoic Acid Receptors (ACTR)

ACTR is a crucial oncogenic factor in HCC. It is also significantly elevated in sorafenib-resistant HCC cells in mice transplant models of HCC, increasing sorafenib resistance by modulating the Warburg effect. Cancer cells produce energy mainly through glycolysis. ACTR not only interacts with c-myc, a key regulator of the Warburg effect, to promote glycolysis to occur, but also promotes glucose uptake, ATP and lactate production, decreases extracellular acidification and oxygen consumption, thus inhibiting sorafenib sensitivity. Knockdown of ACTR decreases the expression of glycolytic enzyme and is associated with a better prognosis (17). Hence, ACTR is expected to be a prospective target for reversing sorafenib resistance.

#### Autophagy

Autophagy exerts a protective effect on tumor cells, and the EFGR/Ras/MAPK pathway, mammalian target of rapamycin (mTOR) pathway, p53 pathway and HIF-1 signaling pathway are several pathways that regulate autophagy in cancer cells (82). Inactivation of the mTOR pathway has been reported to induce resistance to sorafenib in HCC cells. P70S6K and 4E-BP1 are downstream proteins of mTORC1, and their activity can be inhibited by sorafenib in turn (83). The main upstream inducer of mTORC1 is the PI3K/AKT pathway, and inhibition of AKT reverses the acquired sorafenib resistance in HCC patients, converting protective autophagy into cell death. Moreover, PSMD10 translocates into nucleus and binds to heat shock transcription factor (HSF1), initiating ATG7 transcription, increasing autophagy, and promoting sorafenib resistance. This is a hallmark of poor outcome in HCC patients (84). In addition, N6-methyladenosine (M6A)-modified FoxO3 mRNA is downregulated in hypoxic environments, activating autophagy and promoting sorafenib resistance during HCC treatment (85). In conclusion, autophagy is a self-protective mode of HCC cells and a facilitator of sorafenib resistance. Regulating the upstream and downstream pathways of autophagy and converting protective autophagy into apoptosis may be effective approaches for HCC therapy.

#### Epithelial-Mesenchymal Transition (EMT)

EMT is known to be a key process in cancer development, promoting cell migration, and is also associated with resistance to sorafenib. There may be a negative correlation between EMT and the efficacy of sorafenib (86). Galactosin-1 induces EMT in HCC through activating FAK/PI3K/AKT signaling pathway to enhance sorafenib resistance and is a biomarker for predicting sorafenib sensitivity. Sorafenib inhibits the occurrence of EMT, which in turn impairs the efficacy of sorafenib. It has been shown that zinc finger protein 703 may be a promising target for cancer therapy by directly binding to and transfecting the CLDN4 promoter to activate the expression of CLDN4, inducing EMT and inhibiting the sensitivity of HCC cells to sorafenib (87).

The above-mentioned resistance mechanisms of sorafenib can help us to have a deeper understanding of the reasons for the poor chemotherapeutic effect of HCC, and to explore more targets to reverse the resistance of sorafenib for better therapeutic effect.

## MiRNAs and Sorafenib Resistance of HCC

miRNAs are a category of short non-coding RNAs (about 20nt) that bind to the 3’-untranslated region (3’ UTR) of mRNA, act as mRNA sponges to absorb mRNA and regulate the expression levels of downstream genes, participate in several physiological processes, influence cancer-associated pathways, and also contribute to the formation of the resistance to sorafenib (**Table 1**) (83).

### Acting on Autophagy

MiR-423-5p is closely associated with treatment sensitivity. Statistical analysis revealed an increase in secretion of miR423-5p in 75% of patients after 6 months of sorafenib treatment. Further experiments has showed that the proportion of cells in the S phase of the cell cycle in HCC cells is markedly elevated after transfection with miR-423-5p (10). miR-423-5p has been revealed to promote autophagy and ultimately induce drug resistance by targeting growth arrest and DNA damage inducing β protein (GADD45B) (11). miR-142-3p is a tumor inhibitor miRNA that suppresses HCC cell invasion and migration by repressing the expression of the high mobility histone B1 (HMGB1) gene (12). It has also been shown to be an autophagy-regulating miRNA. miR-142-3p upregulation can decrease autophagy by targeting autophagy-related 5 (ATG5) and autophagy-related 16-like 1 (ATG16L1), thereby increasing the sensitivity of HCC cells to sorafenib and enhancing the apoptosis induced by sorafenib. PU.1 transcription factor can up-regulate the miR-142-3p expression. Targeting the PU.1-miR-142-3p-ATG5/ATG16L1 axis may be an effective therapeutic strategy to reverse cytoprotective autophagy and overcome sorafenib resistance (12, 88).

### Acting on Apoptosis

Overexpression of miR-221 activates caspase-3 thereby reversing the resistance of HCC cells to sorafenib and inducing apoptosis in HCC cells. In sorafenib-resistant cells, the activation of insulin-like growth factor 1 receptor (IGF-1R) triggered downstream EGFR pathway RAS/RAF/ERK signaling, resulting in the reduced miR-221 expression and poor therapeutic efficacy (14). Besides, miR−223 targets FBW7 to enhance the resistance of HCC cells to sorafenib and inhibit apoptosis. In addition, liver tumor-initiating cells (T-ICs) play a vital part in the occurrence, development, drug resistance and recurrence of HCC (32). In liver T-ICs, miR-96 down-regulates TP53INP1, inhibits HCC cell apoptosis, and promotes HCC cell resistance to sorafenib (34). Furthermore, enhancers of zeste 2 multimeric complex 2 subunit (EZH2) inhibit HCC cell apoptosis and promote sorafenib resistance by suppressing the expression of miR-101, miR-122, miR-125b and miR-139 thereby regulating insulin-like growth factor 1 receptor (IGF1R) levels (39). In HBV-positive HCC cells, MCL-1 level is elevated and miR-193b is markedly down-regulated. Upregulation of miR-193b can restore cellular sensitivity to sorafenib and promote sorafenib-induced apoptosis (40). Moreover, NRF-2/miR-1 axis-regulated programmed death ligand-1 (PD-L1) inhibits apoptosis of HCC cells, enhances sorafenib resistance and promotes tumor progression (39).

### Acting on AKT Activation

MiR-30a-5p expression is decreased in HCC tissues, inhibits the PI3K/AKT axis, targets CLCF1, and then increases the sensitivity of HCC cells to sorafenib (20). MiR-375, which is also down-regulated in HCC, inhibits AKT activation by targeting AEG-1, suppresses tumor angiogenesis in HCC, and reverses the sorafenib resistance of HCC (31). miR-486-3p, similarly down-regulated, activates AKT by targeting FGFR4 and EGFR, leading to the sorafenib resistance in HCC patients (17). Down-regulation of miR-222 significantly inhibits the proliferation, migration and invasion of HepG2 cells (human hepatocellular cancer cell line, as HCC model) (89) and induces apoptosis. It regulates the expression of phosphorylated PI3K and AKT, thereby enhancing the sorafenib resistance of HCC (25). Moreover, miR-552 promotes hepatic T-IC amplification and decreases the sensitivity of HCC cells to sorafenib. It acts by targeting PTEN to affect AKT activation (22). Furthermore, exosome miR-21 modulates the TETS/PTENP1/PTEN pathway to facilitate the development of HCC, and also inhibits autophagy-mediated sorafenib resistance *via* the PTEN/AKT pathway (29, 30). Besides, miR-494 is related to stem cell phenotypes and promotes the progression of HCC. It enhances the sorafenib resistance of HCC cells *via* targeting PTEN, a crucial protein that inhibits the activation of AKT (35).

### Other Pathways

MiR-30e-3p affects tumor development *via* the MDM2/TP53 axis, and EpCAM, PTEN and P27 are its additional targets that together exert miR-30e-3p’s role in promoting tumor malignancy. In sorafenib-treated HCC patients, increased circulating miR-30e-3p levels predict the progression of sorafenib resistance (16). Likewise, miR-181a promote sorafenib resistance. Studies have reported that HepG2 cells are more sensitive to sorafenib than Hep3B cells and that miR-181a expression levels are lower in HepG2 cells. miR-181a directly targets and reduces the expression of MAPK signaling factor, RASSF1, an inhibitor of sorafenib resistance, thereby reducing sorafenib efficacy (24). Furthermore, miR-216a/217 expression level is increased in HCC and induces EMT *via* targeting PTEN and Smad7, leading to sorafenib resistance and cancer recurrence (43).

The above are miRNAs that promote sorafenib resistance, miRNAs that enhance sorafenib sensitivity is introduced in this paragraph. First, inhibitors of EZH2 can target Notch1 through Notch1-related miRNAs, such as miR-21-5p, miR-26a-1-5p, and act on the corresponding HCC stem cells to reduce sorafenib resistance (37). Second, in most HCC patients, miR-122 is down-regulated. It has been shown that miR-122 targets SERPINB3 and its low expression is related to SERPINB3 activity and CSC phenotype in HCC cells. Moreover, miR-122 have a crucial action in inhibiting sorafenib resistance by regulating the expression of HIF-2α (18). Third, miR-1226-3p is lowly expressed in HCC cells, and it promotes the sensitivity of HCC cells to sorafenib by downregulating DUSP4 and affecting the JNK-Bcl-2 pathway (21). Fourth, miR-124 acts on CAV1 to regulate HCC CSC proliferation, thereby inhibiting sorafenib resistance (23). Fifth, a study has found that LXR increases the sensitivity of sorafenib in HCC by activating miR-378a transcription. MiR-378a is downregulated in HCC, it targets IGF-1R and inhibits EMT, thereby suppressing sorafenib resistance (26). Sixth, miR-744 affects the expression of PAX2, thus inhibiting the multiplication of HCC cells and sorafenib resistance (27). Seventh, miR-374b inhibits the progression of HCC and re-sensitizes HCC cells to sorafenib through antagonizing the PKM2-related glycolysis pathways (28). Eighth, downregulation of miR-145-5p and promoter hypomethylation mediated HDAC11 overexpression affects the metabolism of HCC cells and tissues, and facilitates the metastasis of HCC cells and their resistance to sorafenib (33). Ninth, miR-3609 retards the sorafenib clearance in HCC cells through regulating EPAS-1 and inhibiting activation of the gestation hormone X receptor pathway (38). Last but not least, miR-137 upregulation reverses CSC phenotype and sorafenib resistance in HCC by degrading ANT2 (42).

## LncRNAs and Sorafenib Resistance of HCC

LncRNA is a class of RNAs, over 200 nucleotides in length, with no protein-coding action. It can not only act as a sponge for a variety of miRNAs, but also interact with one or more RNA-binding proteins (RBPs) to be involved in multiple biological processes by regulating cell proliferation, apoptosis, metastasis, and invasion (90, 91). In recent years, its effects on the occurrence, migration, prognosis, recurrence and chemoresistance of cancers have become a hotspot of research (**Table 2**).

### Acting on Autophagy

LncHANR expression is increased in HCC. It serves as a sponge for miR-29b ang inhibits its expression, thus affecting the expression of autophagy-related protein 9A antibody (ATG9A), the target protein of miR-29b, and ultimately enhancing autophagy-associated sorafenib resistance (45). LncNEAT1 can promote the development of multiple cancers including HCC. lncNEAT1 serves as a sponge for miR-204, upregulates ATG3 expression, a target gene of miR-204, promotes autophagy, and facilitates the sorafenib resistance of HCC (57).

### Acting on AKT Activation

LncSNHG1 is remarkably upregulated in HCC tissues and cells, promoting HCC invasion and leading to poor patient prognosis. Further mechanistic studies revealed that sorafenib can induce miR-21 translocation to the nucleus, which promotes lncSNHG1 expression, thereby upregulating solute carrier family 3 member 2 (SLC3A2) leading to AKT activation and ultimately to sorafenib resistance (92). Conversely, downregulation of SNHG1 enhances the effect of sorafenib. LncNEAT1, which also plays a pro-oncogenic role, is significantly increased in HCC cells. It inhibits the efficacy of sorafenib by targeting miR-149-5p and regulating the miR-149-5p/AKT1 axis (47). Furthermore, NEAT1 inhibits the sensitivity of HCC cells to sorafenib *via* modulating miR-335/c-Met (58). In addition, lncHEIH is also markedly upregulated in sorafenib-resistant HCC cells. HEIH acts as a sponge for miR-98-5p to activate the PI3K/AKT pathway, thereby enhancing sorafenib resistance (48). Besides, lncTTN-AS1 expression is upregulated in HCC cells, and lncTTN-AS1 acts as a sponge for miR-16-5p to inhibit its expression, thereby upregulating cyclinE1, the target protein of miR-16-5p, and activating the PTEM/AKT signaling pathway, ultimately leading to resistance of HCC cells to sorafenib (93).

### Acting on Apoptosis

LncFAM225A is up-regulated in HCC tissues and sorafenib-resistant HepG2/SOR cells, and inhibition of FAM225A significantly inhibits the resistance of HepG2/SOR cells to sorafenib. Further studies have revealed that FAM225A interacts with miR-130a-5p to negatively regulate CNG1 expression, thereby inhibiting apoptosis and promoting sorafenib resistance of HCC cells (46). Besides, lncKCNQ1OT1 has been revealed to correlate with the sorafenib resistance and immune escape of HCC cells. In HCC tissues resistant to sorafenib, KCNQ1OT1 serves as a ceRNA for miR-506 and elevates the expression of PD-L1, leading to immune escape of HCC cells. Knockdown of KCNQ1OT1 could alter the tumor microenvironment, inhibit T-cell apoptosis and promote HCC cell apoptosis, thus inhibiting HCC cell resistance to sorafenib and cell metastasis (51). Furthermore, by restraining miR-335 expression, lncNCNEAT1 suppresses c-Met-AKT signaling pathway-mediated sorafenib resistance of HCC cells. In contrast, upregulation of miR-335 or knockdown of c-Met resists the antiapoptotic activity of NEAT1 in HCC cells (58).

### Acting on EMT

LncSNHG16 is closely associated with HCC invasiveness and poor outcome of patients, and its expression is dramatically increased in HCC cells. Additionally, SNHG16 can serve as an endogenous sponge of miR-140-5p and up-regulate flap endonuclease 1 (FeN1), an oncogene in a range of cancers, which is also involved in the pathological process of HCC, thereby reducing the sensitivity of HCC cells to sorafenib and affecting the therapeutic effect (49). Previous study has confirmed that silencing FeN1 restrains the EMT of HCC cells, thereby suppressing HCC progression and metastasis (50). In summary, SNHG16 regulates the EMT of HCC cells through affecting the miR-140-5p/FeN1 axis, thereby promoting the sorafenib resistance of HCC.

The lncMALAT1 expression is increased in sorafenib-resistant HCC tissues, and enhances the multiplication, migration and EMT of HCC cells, thus affecting the development and progression of HCC. It has shown that MALAT1 as a sponge for miR-140-5p and promotes the expression of the serine/threonine protein kinase Aurora-A, which maintains genomic integrity and participate mitosis. Up-regulated Aurora-A is related to poor outcome of HCC patients and induces a variety of malignant phenotypes in HCC (52, 53). In addition, lncH19 expression is negatively related to sensitivity of HCC cells to sorafenib. Knockdown of lncH19 can improve the sensitivity of HCC cells to sorafenib by inhibiting EMT (54). Notably, H19 can upregulate the expression of miR-675 to promote EMT. What’s more, through serving as a sponge for miR-182-5p, lncPOIR inhibits the expression of miR-182-5p and promotes EMT, thereby suppressing sorafenib sensitivity and promoting HCC development. Knockdown of lncPOIR reverses the EMT and the sorafenib resistance of HCC cells (59).

### Acting on Other Pathways

It is shown that LncFOXD2-AS1 is significantly reduced in HCC cells. FOXD2-AS1 affects the transmembrane protein 9 (TMEM9) by suppressing miR-150-5p expression, thereby reversing sorafenib resistance in HCC (55). In contrast, lncTUC338 is highly expressed in HCC cells and tissues. Down-regulation of TUC338 suppresses tumor growth by increasing the expression of Rasal1, while enhancing the sensitivity of HCC cells to sorafenib *via* inhibiting gluconeogenesis (60, 61). Knockdown of lncVLDLR reduces the expression of ABCG2, an important transporter, thereby restraining drug efflux and enhancing sensitivity of HCC cells to sorafenib. lncVLDLR may be a novel target to improve the efficacy of sorafenib (62).

## CircRNAs and Sorafenib Resistance of HCC

CircRNAs are a specific category of ncRNAs and a latest research hotspot in the field of RNA. Unlike linear RNAs, circRNAs, with a closed loop structure, cannot be sheared by RNA exonucleases and are more stably expressed and less susceptible to degradation. They widely correlate with the regulation of cell multiplication, polarization and apoptosis *in vivo*, and also have significant effects on the development of various diseases, particularly on the pathogenesis, diagnosis, treatment and prognosis of tumors (94). However, studies on the role of circRNAs in the sorafenib resistance of HCC are still in the infancy. It has been found 582 differentially expressed circRNAs in HCC cells resistant to sorafenib (HUH7-S), of which 272 were up-regulated and 310 were down-regulated, with a statistically significant difference (0.05) (95). This suggests that circRNAs with different expression levels may exert a crucial role in sorafenib resistance of HCC.

N6-methyladenosine (M6A)-modified circRNA-SORE is expressed highly in sorafenib-resistant HCC cells. Further studies have shown that circRNA-SORE can induce EMT through serving as a sponge for miR-103a-2-5p and miR-660-3p to competitively activate Wnt/β-catenin pathway, thereby inducing sorafenib resistance in HCC cells (15). Furthermore, circRNA-SORE can bind to YBX1, a major oncogenic protein in cytoplasmic matrix, thereby preventing YBX1 from interacting with E3 ubiquitin ligase PRP19, blocking PRP19-mediated degradation of YBX1, stabilizing YBX1, and ultimately mediating sorafenib resistance in HCC cells and poor prognosis of patients (96).

CirCFN11 expression is increased in HCC cells, and its overexpression promotes the aggressiveness of HCC and is an independent risk factor for prognosis of HCC patients. It acts as a sponge of miR-1205 to upregulate the expression of oncogene E2F1 and finally mediates the resistance of HCC cells to sorafenib (97).

Moreover, circFoxM1, a newly discovered circRNA, significantly inhibits HCC development and enhances sorafenib efficacy *in vitro*. Interestingly, its expression is up-regulated in sorafenib-resistant HCC cells, suggesting that circFoxM1 may affect the development of HCC and sorafenib resistance, respectively, through different mechanisms. CircFOXM1 can serve as a sponge for miR-1324 and increase methyl-CpG-binding protein 2 (MeCP2) expression, thereby regulating sorafenib resistance in HCC. In addition, overexpression of miR-1324 reverses circFoxM1-induced sorafenib resistance and increases MeCP2 expression. This further confirms that circFOXM1 contribute to the regulation of sorafenib sensitivity in HCC cells through the miR-1324/MeCP2 axis (95).

## Conclusions and Expectations

A growing number of ncRNAs have been identified to play critical regulatory roles in sorafenib resistance of HCC. MiRNAs exert regulatory effects by binding to the 3’-UTR of target mRNAs to regulate downstream proteins, while lncRNAs and circRNAs are mainly involved in modulating the resistance of sorafenib as sponges of miRNAs.

The underlying mechanisms of the role of sorafenib resistance-associated ncRNAs in HCC are summarized in **Figure 1**. Targeting these dysregulated ncRNAs may be a promising approach to reverse sorafenib resistance in HCC. Delivery of tumor suppressor ncRNAs directly or *via* suitable vectors to target cells to exogenously increase their expression, or designing small interfering RNA (siRNA) or short hairpin RNA (shRNA) to knock down oncogenic ncRNAs has been shown to be adaptable in reversing resistance to sorafenib in HCC patients. For example, gold nanoparticles-loaded anti-miR221 has been shown to enhance the effect of sorafenib by inhibiting HCC cell proliferation *via* inactivating miR-221/p27/DNMT1 pathway. In addition, the treatment with the combination of nanoparticles-loaded anti-miR221 and sorafenib is more efficient than with sorafenib alone (98). It has also been reported that miR-375 and sorafenib can be co-loaded into calcium carbonate nanoparticles with lipid coating to inhibit the resistance of sorafenib *via* exerting the anti-autophagic effect of miR-375, thus enhancing the anti-tumor effect of sorafenib (99). Matrine combined with sorafenib treatment also inhibits proliferation of HCC cells synergistically, partially by suppressing miRNA-21 expression and then inducing PTEN (100). Hence, targeting ncRNAs in combination with sorafenib against HCC is expected to conquer sorafenib resistance and represent a promising option for patients in advanced stage of HCC resistant to sorafenib. However, it is still a challenge to select key target ncRNAs from numerous candidate ncRNAs. In order to develop ncRNA-based therapeutics to benefit HCC patients, further additional translational research and clinical trials are warrant. We believe that targeting ncRNAs is promising to eventually overcome sorafenib resistance thus improving outcome of advanced HCC patients.

It is worth noting that the development of more reliable delivery systems is urgently required to improve the biological stability of ncRNAs and to improve their uptake, penetration, transport, distribution and retention. Meng et al. constructed 50 nm mesoporous silica nanoparticles to transport doxorubicin and Pgp siRNA, which are protected *via* a polyethyleneimine-polyethylene glycol copolymer, resulting in an 8% increase in doxorubicin and Pgp siRNA permeability by 8%, ultimately reversing the resistance of breast cancer to doxorubicin. This practice provides a rationale for ncRNAs-based therapy combined with sorafenib to against sorafenib resistance in HCC and improve patients’ prognosis (101). Development of suitable transport vehicles for ncRNA/siRNA/shRNA and sorafenib is important to improve combination therapy to reverse sorafenib resistance and improve sorafenib efficacy.

## Author Contributions

All the authors made substantial contributions to this study. All authors contributed to the article and approved the submitted version.

## Funding

This work was supported by the National Natural Science Foundation of China (no. 31971166 to XX).

## Conflict of Interest

The authors declare that the research was conducted in the absence of any commercial or financial relationships that could be construed as a potential conflict of interest.
